# Modelling the transmission dynamics of severe fever with thrombocytopenia syndrome in Jiangsu Province, China

**DOI:** 10.1186/s13071-021-04732-3

**Published:** 2021-05-06

**Authors:** Nan Zhang, Xiao-Qing Cheng, Bin Deng, Jia Rui, Luxia Qiu, Zeyu Zhao, Shengnan Lin, Xingchun Liu, Jingwen Xu, Yao Wang, Meng Yang, Yuanzhao Zhu, Jiefeng Huang, Chan Liu, Weikang Liu, Li Luo, Zhuoyang Li, Peihua Li, Tianlong Yang, Zhi-Feng Li, Shu-Yi Liang, Xiao-Chen Wang, Jian-Li Hu, Tianmu Chen

**Affiliations:** 1 Department of Acute Infectious Diseases Control and Prevention, Jiangsu Provincial Centre for Disease Control and Prevention, 172, Jiangsu Rd, Nanjing, 210009 China; 2 State Key Laboratory of Molecular Vaccinology and Molecular Diagnostics, School of Public Health, Xiamen University, Xiamen, 361102 Fujian People’s Republic of China

**Keywords:** Severe fever with thrombocytopenia syndrome, Bunyavirus, Mathematical model, Environment, Ticks, Transmission, Dynamic

## Abstract

**Background:**

Severe fever with thrombocytopenia syndrome (SFTS) is an emerging infectious disease that is regionally distributed in Asia, with high fatality. Constructing the transmission model of SFTS could help provide clues for disease control and fill the gap in research on SFTS models.

**Methods:**

We built an SFTS transmission dynamics model based on the susceptible–exposed–infectious–asymptomatic–recovered (SEIAR) model and the epidemiological characteristics of SFTS in Jiangsu Province. This model was used to evaluate the effect by cutting off different transmission routes and taking different interventions into account, to offer clues for disease prevention and control.

**Results:**

The transmission model fits the reported data well with a minimum *R*^2^ value of 0.29 and a maximum value of 0.80, *P* < 0.05. Meanwhile, cutting off the environmental transmission route had the greatest effect on the prevention and control of SFTS, while isolation and shortening the course of the disease did not have much effect.

**Conclusions:**

The model we have built can be used to simulate the transmission of SFTS to help inform disease control. It is noteworthy that cutting off the environment-to-humans transmission route in the model had the greatest effect on SFTS prevention and control.

**Graphical Abstract:**

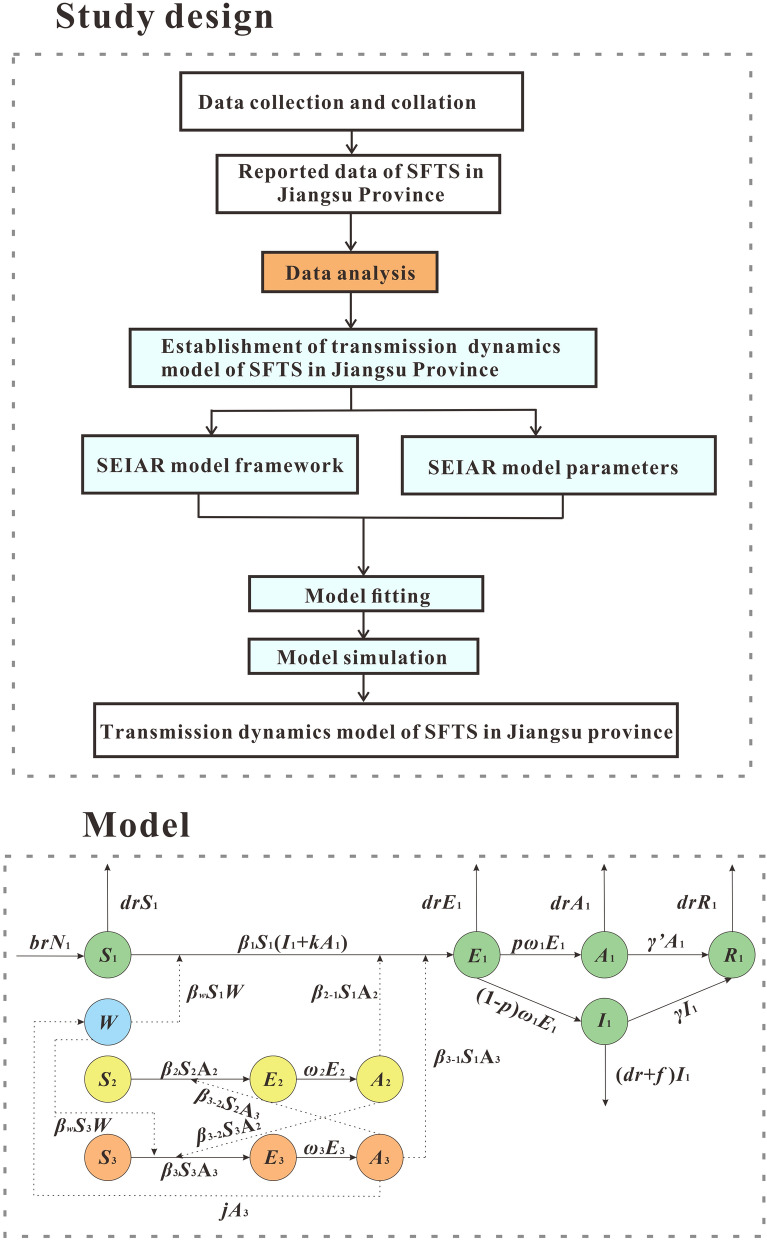

**Supplementary Information:**

The online version contains supplementary material available at 10.1186/s13071-021-04732-3.

## Background

Severe fever with thrombocytopenia syndrome (SFTS) is an emerging infectious disease caused by a novel bunya virus called severe fever with thrombocytopenia syndrome virus (SFTSV) [1]. The clinical manifestations of infection in human are characterized by high fever, a drastic reduction of platelets and leukocytes and hemorrhage [2–5]. The clinical course is mainly divided into three stages: fever, multiple nerve dysfunction and recovery [1, 3, 4, 3, 7].

In previous years, SFTS occurred mainly in the rural areas of central and northeast China, with an estimated 500–1500 cases recorded in 2011–2012. Over time, SFTS has spread to South Korea and Japan, although the related fatality rate of nearly 30% has dropped to 10–12% in recent years [8]. In South Korea, the fatality rate was above 35% among infected patients in 2012–2013, but the incidence rate dropped to 11.52% in 2016 [9]. In Japan, however, the fatality rate is slightly higher than that observed in China (23%), and cases have mainly occurred in the western regions [10]. Currently, the SFTS fatality rate in Asia seems to be decreasing, while the incidence rate is increasing regionally, especially in China. According to the monitoring data from the China Information Network System of Disease Prevention and Control, SFTS incidence and prevalence rates have increased each year from 2010 to 2016, with cases found in 23 provinces [11]. Analyzing the epidemiological characteristics of SFTS, one published study found Henan Province, Hubei Province and Anhui Province to be the main provinces with the largest numbers of cases before 2011 [12]. Including five other provinces, SFTS cases were detected in Jiangsu Province as far back as 2011 [13]. Since then, the number of SFTS cases reported in Jiangsu Province has increased annually, with more than 140 confirmed cases in over 60 locations within the province by 2016 [14].

To fight against this emerging infectious disease, findings from both domestic and foreign research on the etiology and risk factors of the disease are of great importance. As shown by these study findings, SFTS mainly occurred in tick-active months from May to September, and reports of new infections continued until November. The main population affected by SFTS is farmers aged 50 years old and above who live in forested or hilly areas [15–18]. Recent researches suggested that ticks are the main host of SFTS and play a key role in its transmission. *Haemaphysalis longicornis*, *Ixodes nipponensis*, *Rhipicephalus microplus* and *Dermacentor sinicus*, amongst others, are the susceptible ticks identified, but *Haemaphysalis longicornis* is the dominant species [1, 19–22]. While SFTS cases occurred from March to November, the disease period peaked in August and September. The seasonal distribution of SFTS cases was also found to be closely related to the seasonal trend of tick density [23, 24]. Some preliminary researches exist to show the process of host animal–tick–human natural transmission routes of SFTS [25]. However, there are very few systematic and definite researches on transmission routes among vector, host and SFTSV-affected populations. Meanwhile, various problems exist in the treatment of SFTS such as unclear pathogenesis, absence of vaccine for prevention and lack of a specific SFTS antiviral therapy. As a result, a low fatality rate cannot be maintained.

SFTS infection also induces a certain amount of economic pressure on affected populations, as a study has shown that patients and their families pay nearly $3158.4 for treatment [2]. This makes SFTS a significant threat to public health, as the treatment amount is almost equal to the disposable income of rural residents in Anhui Province as of 2017 [2]. To date, no research exists on a transmission dynamics model of SFTS, and there is no clear evidence on preventive methods that could help achieve optimal intervention effects.

This study aimed to construct a multi-population and multi-route dynamic model (MMDM) based on the susceptible–exposed–infectious–asymptomatic–recovered (SEIAR) model, the epidemiological characteristics and the transmission features of SFTS in Jiangsu Province.

## Methods

### Study area

Jiangsu Province is located between 30°45′ and 35°20′ north latitude and 116°18′ to 121°57′ east longitude (Additional file 1: Figure S1). Jiangsu Province is located in the East Asian monsoon climate zone, in the transitional zone between subtropical and warm temperate climate. The total area of Jiangsu Province is 107,200 square kilometers, with an estimated permanent population of 80.7 million as of the end of 2019.

## Study design

**Fig. 1:**
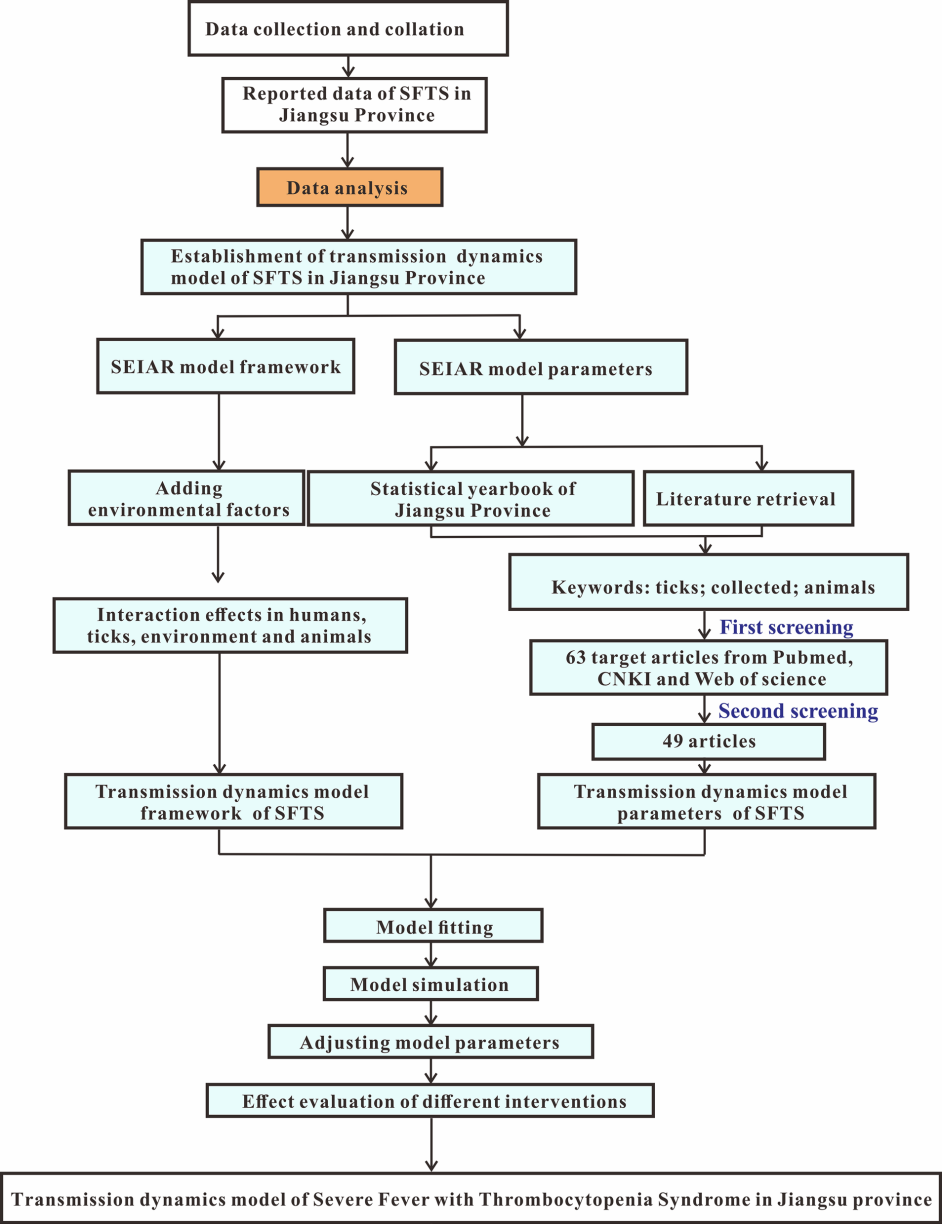
Flowchart of study design

This study was designed by three steps. First step, the MMDM model of SFTS was established by searching scientific literature and analyzing reported data. Second step, the reported data of SFTS in Jiangsu Province from January 2001 to December 2019 was used to fit the developed model. Third step, the MMDM model was used to simulate the intervention effect of eliminating different routes of transmission. This was achieved by setting the parameters of the model to obtain the best intervention plan, thus providing clues to effective disease control interventions and filling the gap in the research on SFTS models (Fig. 1).

### Establishment of transmission dynamics model

**Fig. 2:**
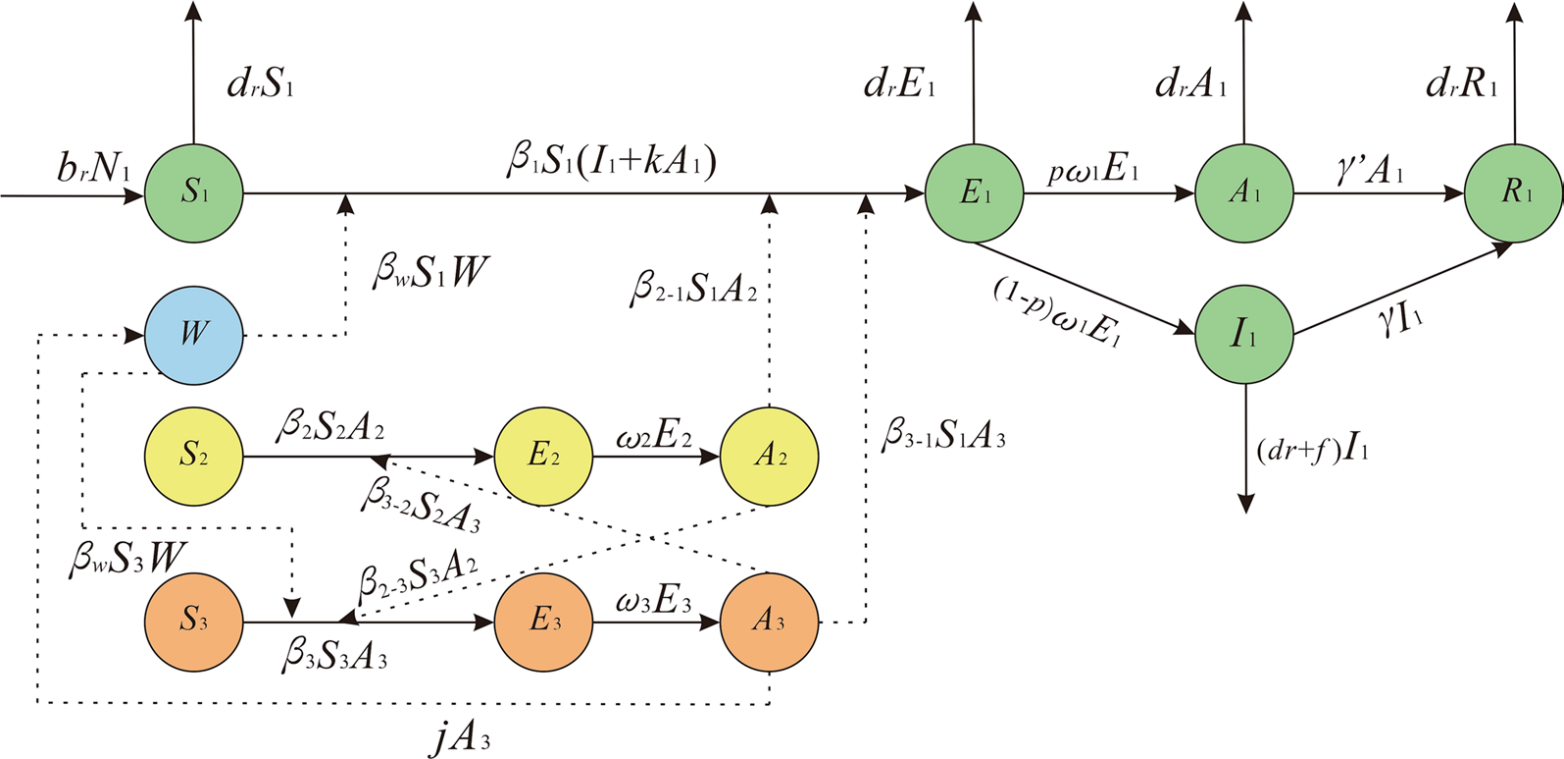
Flowchart of the MMDM without intervention

Based on the characteristics of epidemic and the routes of transmission of SFTS in Jiangsu Province and the SEIAR model [14, 26–28], this study established the MMDM to simulate SFTS transmission. This model included four parts, namely human, ticks, host animals and environment, which are represented by subscript 1, 2, 3 and *w*, respectively (Fig. 2).

### Human part of the MMDM

In this part, humans were categorized as: susceptible (*S*_1_), exposed (*E*_1_), asymptomatic (*A*_1_), infectious (*I*_1_) or recovered/remover (*R*_1_). The structure of the MMDM is shown in Fig. 2, and this part of the MMDM was based on the following assumptions:

1. This model supposed that all human newborns are susceptible to the disease. Under this assumption, the total population is denoted by *N*, the birth rate is *br* and the natural mortality rate is *dr*. According to the literature, the fatality rate of overt infections, *f*, is 16.1% [5]. Meanwhile, this model took the population-wide mortality rate to approximately replace the mortality rate of the excluded overt infections.
2. According to the literature, people can be infected with SFTS by coming in contact with the blood of an infected patient [29]. At the same time, the transmissibility of *I*_1_ and *A*_1_ are different. Therefore, we assumed that the transmissibility of *A*_1_ is *κ* times that of *I*_1_ *κ*. The infection rate coefficients of *I*_1_ and *A*_1_ are *β*_1_ and *κβ*_1_, respectively.
3. We assumed that certain percentage (1 − *p*) (0 ≤ *p* ≤ 1) of *E*_1_ will transform into *I*_1_ after an incubation period, while the other part (*p*) of *E*_1_ will become *A*_1_ after a latent period. Hence, at time *t*, the development speed of *E*_1_ to the *I*_1_ pathway is proportional to the number of people in the incubation period, and the proportional coefficient is (1 − *p*) *ω*_1_. The development speed of *E*_1_ to the *A*_1_ pathway is proportional to the number of people in the latent period, and the proportional coefficient is *pω*_1_.
4. The out speed of the population in the *I*_1_ and *A*_1_ groups is respectively proportional to their population number, and the ratio coefficients are respectively *γ* and *γ*'.
5. The infectors of SFTS will produce antibodies after they are removed, but this study supposed that the *R*_1_ will never be infected and that the *R*_1_ is the destination of the transmission route between people, because literature on a second infection in the population was not found.

### Environment part of the MMDM

In this part, *W* represent the environmental transmission route in the MMDM, and this part was based on the following assumptions:

- 1. Although no literature strongly proved that the environment is one of the transmission route, some evidence suggested that the possibility of getting SFTS infection SFTS in the environment was higher than by ticks [30–33]. Hence, we assume that *W* has the ability to facilitate the spread of SFTS to humans and animals. We set the transmissibility of *W* to animals and human as *β*_*w*_ [34].
- 2. As reported, the blood and excreta of infected human or host animals may diffuse the SFTSV. And as SFTS has commonly occurred extensively in the wild, we assumed that animals have the possibility to infect the environment by their blood or excreta. We set the propagation coefficient *j* as 10 [35].

### Tick part of the MMDM

In this part, the ticks were divided into susceptible ticks (*S*_2_), exposed ticks (*E*_2_) and asymptomatic ticks (*A*_2_). And this part of the MMDM was based on the following assumptions:

1. According to literature, SFTS mainly spreads by ticks [1, 19–21], and infected ticks can spread the virus by biting host animals or humans [36].
2. As the long-term storage host, ticks infected with SFTSV can preserve the pathogen and retain the infectious ability of the pathogen without symptoms [36]. So this study set the transmission route among ticks as the susceptible–exposed–asymptomatic (SEA) model.
3. In transmission among ticks, *A*_2_ has transmissibility to infect ticks, and the infection rate coefficient is *β*_2_, but our study considered that the SFTS was not diffused among ticks, so we set *β*_2_ as 0. And the *S*_2_ will become *E*_2_ by biting the infected animals and humans.
4. This study supposed that after one latent period, all the *E*_2_ will transform to *A*_2_. So at time *t*, the number of ticks in the *E*_2_ to *A*_2_ route is *ω*_2_*E*_2_.
5. Ticks in the state of latent infection can infect host animals through biting. As the coefficient of infection rate of the virus spreading from ticks to a host animal is *β*_2-3_, we denoted the part of host animal infection by a tick’s bite as *β*_2-3_*S*_3_*A*_2_. In addition, ticks can also spread SFTS among humans through direct bites. Since the coefficient of infection rate of the virus spreading from ticks to humans is *β*_2-1,_ the part of humans getting the virus by tick bites is denoted as *β*_2-1_*S*_1_*A*_2_.

### Host animal part of the MMDM

In this part, the animals were divided into susceptible animals (*S*_3_), exposed animals (*E*_3_) and asymptomatic animals (*A*_3_). The assumptions of this part in the MMDM were as follows:

1. According to reports by one study, the main amplification hosts of arboviruses are small rodents. This meant that arthropods can cause relative viremia by biting small rodents. However, other susceptible arthropods can also be infected and make the virus spread further by biting infected small rodents [37].
2. For SFTS, ticks infected with SFTSV can cause viremia by biting host animals. At the same time, susceptible ticks can also be infected by biting infected host animals [37–40]. Therefore, we still set the transmission progress of SFTSV as the SEA model in the host animal part of the transmission dynamics model.
3. Among the transmission of host animals, *A*_3_ has transmissibility that can make *S*_3_ transform to *E*_3_ with the infection rate coefficient *β*_3_. At the time *t*, the number of host animals in the *S*_3_ to *E*_3_ pathway was *β*_3_*S*_3_A_3_.
4. This study supposed that all the *E*_3_ will transform to *A*_3_ after one latent period. At the time *t*, the number of host animals in the *E*_3_ to *A*_3_ pathway was *ω*_3_*E*_3_.
5. The uninfected ticks can be infected after biting the host animal that is in a state of latent infection. The coefficient of host-to-tick transmission of the virus is *β*_3-2_, so ticks with a *β*_3-2_*S*_2_*A*_3_ component can acquire the virus by biting an infected host animal.

### The differential equation

The above MMDM is expressed by the following differential equations:

$$\frac{{dS_{1} }}{dt} = b_{r} N_{1} - d_{r} S_{1} - \beta_{1} S_{1} \left( {I_{1} + kA_{1} } \right) - \beta_{21} S_{1} A_{2} - \beta_{w} S_{1} I_{w} - \beta_{31} S_{1} A_{3}$$

$$\frac{{dE_{1} }}{dt} = \beta_{1} S_{1} \left( {I_{1} + kA_{1} } \right) + \beta_{w} S_{1} I_{w} + \beta_{21} S_{1} A_{2} + \beta_{31} S_{1} A_{3} - d_{r} E_{1} - p\omega_{1} E_{1} - \left( {1 - p} \right)\omega_{1} E_{1}$$

$$\frac{{dA_{1} }}{dt} = p\omega_{1} E_{1} - d_{r} A_{1} - \gamma^{\prime}A_{1}$$

$$\frac{{dI_{1} }}{dt} = \left( {1 - p} \right)\omega_{1} E_{1} - \gamma I_{1} - \left( {d_{r} + f} \right)I_{1}$$

$$\frac{{dR_{1} }}{dt} = \gamma^{\prime}A_{1} + \gamma I_{1} - d_{r} R_{1}$$

$$\frac{dW}{{dt}} = jA_{3}$$

$$\frac{{dS_{2} }}{dt} = - \beta_{2} S_{2} A_{2} - \beta_{32} S_{2} A_{3}$$

$$\frac{{dE_{2} }}{dt} = \beta_{2} S_{2} A_{2} + \beta_{32} S_{2} A_{3} - \omega_{2} E_{2}$$

$$\frac{{dA_{2} }}{dt} = \omega_{2} E_{2}$$

$$\frac{{dS_{3} }}{dt} = - \beta_{w} S_{3} W - \beta_{3} S_{3} A_{3} - \beta_{23} S_{3} A_{2}$$

$$\frac{{dE_{3} }}{dt} = \beta_{3} S_{3} A_{3} + \beta_{23} S_{3} A_{2} + \beta_{w} S_{3} W - \omega_{3} E_{3}$$

$$\frac{{dA_{3} }}{dt} = \omega_{3} E_{3}$$

The left-hand side of the equation was respectively expressed as the instantaneous change velocity of *S*, *E*, *I*, *A* and *R* at time *t*.

### Estimation of parameters

In our MMDM, there were a total of 20 parameters. The values of parameters *p*,1/*ω*_1_,1/*γ*,1/*γ*', *f*,1/*ω*_2_, 1/*ω*_3_ and *j* were all according to relative literatures. Among them, *p* of recessive infection ranged from 3.2% to 5.5% [41], and the mean was 4.3% in this study, i.e., *p* = 0.043. The 1/*ω*_1_ of the incubation period of SFTS in the population ranged from 9 to 14 days [42, 43], and the median was 11 days in this study. The course of the disease 1/*γ* of overt infection was 2 weeks [44], i.e. 14 days. The research about the course of the disease 1/*γ*' of latent infection was not found, so we used the same days with the course of the disease of overt infection, i.e., 14 days. The fatality rate *f* ranged from 11.2% to 30%, and *f* = 0.16 [5]. The incubation period after tick infection with SFTSV was 1/ *ω*_2_ weeks, i.e., 7 days. The incubation period of SFTSV infection in host animals 1/*ω*_3_ was 12 days [39]. No valid data or literature support was found for the relative transmissibility coefficient *κ* of recessive infected persons. In this study, *κ* = 1 was selected for calculation. The *br* and *dr* were obtained from the statistical yearbook of Jiangsu Province, see Table 1.

**Table 1:** Definition and value of parameters

Parameter	Definition	Value	Unit	Method
Person				
*β*_1_	Person-to-person transmissibility coefficient	–	(Person*day)^−1^	Model fitting
*κ*	Relative transmissibility coefficient of unapparent infection	1	1	–
p	Proportion of latent infection	0.043	1	Ref. [41]
1/*ω*_1_	Incubation period	11	Day	Ref. [42]
1/*γ*	Infectious period of dominant infection	14	Day	Ref. [44]
1/*γ*'	Latent infection period	14	Day	–
*f*	Fatality rate	0.16	1	Ref. [5]
*br*	Birth rate	0.009481	1	Statistical Yearbook of Jiangsu Province
*dr*	Mortality rate	0.007003	1	Statistical Yearbook of Jiangsu Province
Ticks				
*β*_2_	Coefficient of transmissibility between ticks	0	(Pieces*day)^−1^	–
*β*_2-1_	Coefficient of tick-to-human transmissibility	8*β*_1_	(Person*day)^−1^	Ref. [34]
*β*_2-3_	Coefficient of tick-to-host infection	8*β*_1_	(Pieces*day)^−1^	Ref. [34]
1/*ω*_2_	Incubation period of ticks	7	Day	Ref. [26]
Host animal				
*β*_3_	Coefficient of transmissibility between host animal	2*β*_1_	(Pieces*day)^−1^	Ref. [34]
*β*_3-1_	Coefficient of transmissibility rate of host animal to human	2*β*_1_	(Pieces*day)^−1^	Ref. [34]
*β*_3-2_	Coefficient of host animal infection to ticks	8*β*_1_	(Person*day)^−1^	Ref. [34]
1/*ω*_3_	Host animal incubation period	12	Day	Ref. [39]
*j*	Rate of host animal discharge to the environment	10	–	Ref. [35]
Environment				
*β*_*w*_	Coefficient of transmissibility rate of the environment to humans and host animals	9*β*_1_	(Human/animal*day)^−1^	Ref. [34]
*W*	Environmental tick density	0.047494291	One km^2^/person (flag hour)	Jiangsu Province surveillance data

The coefficient of infection *β*_1_was generated by model fitting. The Runge–Kutta method of order 4 with tolerance set at 0.001 was used to perform curve fitting of the root mean square deviation between the data and best run so far. In this study, the seasonality of the transmission was considered. According to the MMDM, the seasonality should be dynamic, focusing on *β.* Therefore, a trigonometric function was adopted and shown as follows:

$$\beta { } = \beta_{0} \left[ {1{ } + {\text{ sin}}\left( {\frac{{2\pi \left( {t + \alpha } \right)}}{T}} \right)} \right]$$

In the equation, *β*_0_, *t*, *α* and *T* refer to the baseline of the transmission relative, time, a constant which adjusts the position of time, and the time span of the season cycle, respectively.

### “Knock-out” simulation

“Knock-out” simulation was performed as the reference [45] to quantify cutting different transmission routes. To “knock out” means to cut off the transmission routes between different groups. In the model study, the simulation sets a parameter to 0 and estimates the contribution of the parameter by calculating the number of reduced cases or the total attack rate. For example, in the MMDM, the contribution of environmental transmission *β*_*w*_ was set to 0, and its effect was reflected by calculating the number of reduced cases.

In this study, the transmissibility coefficient in the MMDM was used to reflect different transmission routes, including transmissibility of the environment to humans or host animals (*β*_*w*_), host animals to humans (*β*_3-1_), ticks to humans (*β*_2-1_), humans to humans (*β*_1_), ticks to host animals (*β*_2-3_), host animals to ticks (*β*_3-2_) and host animals to host animals (*β*_3_).

Based on the established SFTS transmissibility model, using 2019 data, a scenario of cutting off various transmission routes was established by setting one of the above infection rates up to seven values or to 0 at the time of simulation. We arranged the matrix between different transmission routes, as shown in Table 2, to perform a more comprehensive re-sectioning of transmission routes. There were seven scenarios at the same time:

Scenario 1: Only one coefficient *β* was controlled to be zero.

we made *β*_*w *= 0_ or *β*_3-1 = 0_ or *β*_2-1 = 0_ or *β*_1 = 0_ or *β*_2-3 = 0_ or *β*_3-2 = 0_ or *β*_3 = 0_. At the same time, we kept the other six *β *coefficients unchanged_._

Scenario 2: Two coefficients *β* were controlled to be zero.

Then, we combined these seven coefficients *β* in pairs so that two of them are equal to zero, while keeping the other five coefficients *β* unchanged.

**Table 2:** Combined transmissibility matrix

	*β*_1_	*β*_3_	*β*_*w*_	*β*_21_	*β*_31_	*β*_32_	*β*_23_
*β*_1_	1	2*β*_1_	9*β*_1_	8*β*_1_	2*β*_1_	8*β*_1_	8*β*_1_
*β*_3_	1/2*β*_3_	1	9/2*β*_3_	4*β*_3_	1*β*_3_	4*β*_3_	4*β*_3_
*β*_*w*_	1/9*β*_*w*_	2/9*β*_*w*_	1	8/9*β*_*w*_	2/9*β*_*w*_	8/9*β*_*w*_	8/9*β*_*w*_
*β*_21_	1/8*β*_21_	1/4*β*_21_	9/8*β*_21_	1	1/4*β*_21_	1*β*_21_	1*β*_21_
*β*_31_	1/2*β*_31_	1*β*_31_	9/2*β*_31_	4*β*_31_	1	4*β*_31_	4*β*_31_
*β*_32_	1/8*β*_32_	1/4*β*_32_	9/8*β*_32_	1*β*_32_	1/4*β*_32_	1	1*β*_32_
*β*_23_	1/8*β*_23_	1/4*β*_23_	9/8*β*_23_	1*β*_23_	1/4*β*_23_	1*β*_23_	1

In the following scenarios, the method adopted was the same as the above two scenarios, that is, the *β* value of the corresponding number was controlled to be zero, and the remaining *β* values remain unchanged. Scenario 3: Three coefficients *β* were controlled to be zero. Scenario 4: Four coefficients *β* were controlled to be zero. Scenario 5: Five coefficients *β* were controlled to be zero. Scenario 6: Six coefficients *β* were controlled to be zero. Scenario 7: Seven coefficients *β* were controlled to be zero.

The above methods were used to obtain the intervention effect of cutting off different transmission routes of SFTS. The indicators of intervention effect included: cumulative number of cases, total attack rate, peak incidence site and peak incidence. This was to provide guidance for the prevention and control of the occurrence of an SFTS epidemic.

### Effectiveness of interventions

**Fig. 3:**
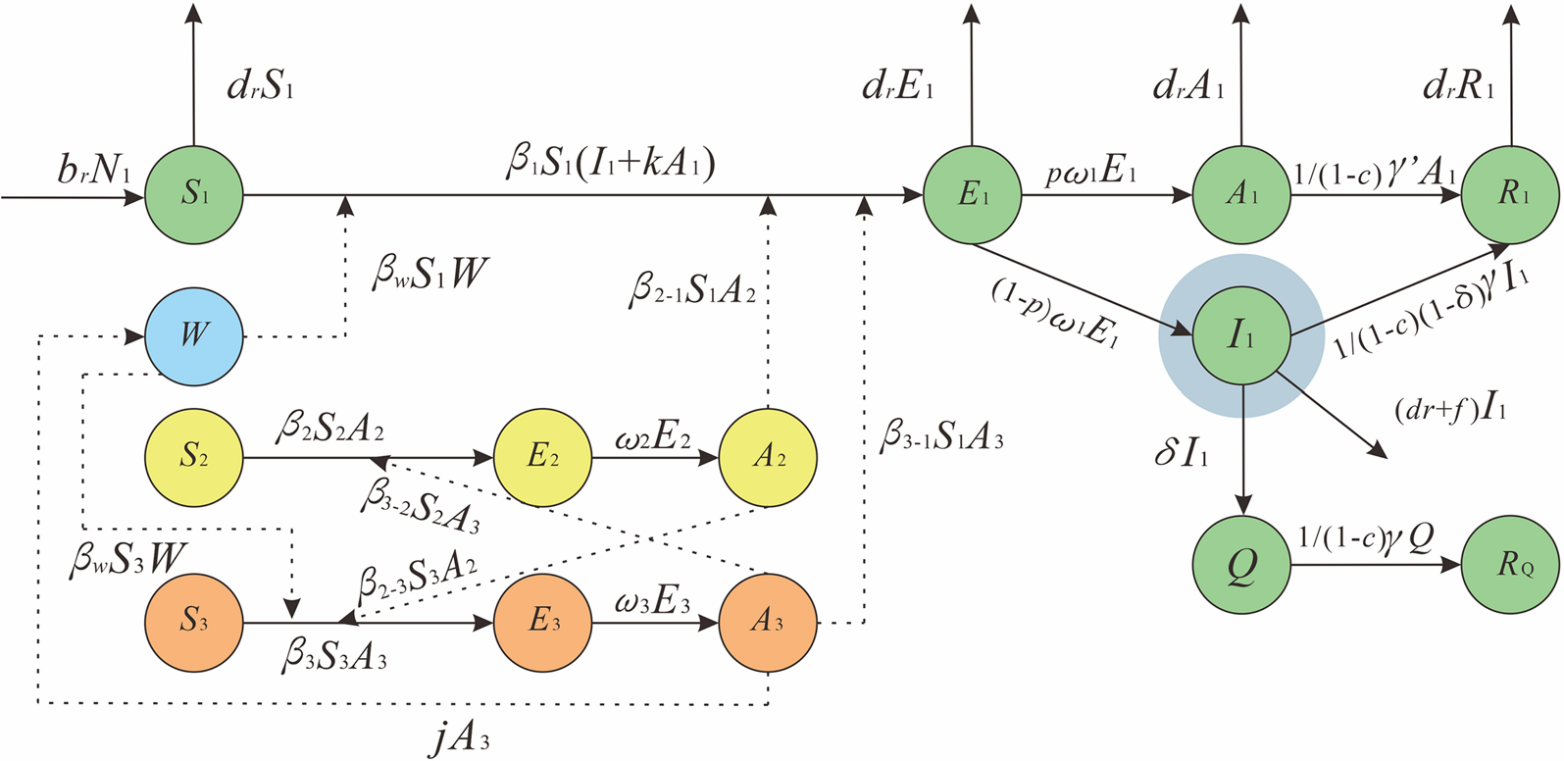
Flowchart of the MMDM with interventions

In this model, we only considered two interventions, and the model was established as shown in Fig. 3. We were trying to isolate the infections according to the transmission route of SFTS from person to person. In addition, although there are no targeted antiviral drugs to treat SFTS, drug treatment can still reduce the mortality rate and increase the clinical recovery rate. Therefore, considering interventions that can shorten the course of the disease, such as hospitalization or drug treatment, was important [46, 47].

For the isolation, *Q* was the number of people isolated, *R*_*Q*_ was the recovery of isolation, *δ* was the isolation coefficient and *c* was the coefficient of shortening the disease course in the model. We set the *ϕ* as the proportion of isolation which can be calculated by $\phi = \frac{{R_{Q} }}{{R_{1} + R_{Q} }}$; we then set *ϕ* as 98%, 90%, 80%, 70%, 60% and 50% to simulate the solo effect of different isolation ratios.

For the intervention to shorten the disease courses, we used *γ* to represent this intervention. We set *γ* as 1/14, 1/12, 1/10, 1/8, 1/6 and 1/4 to simulate the solo effect of shortening different days.

We did not know how many isolated cases and how many days of medication would shorten the course of the disease. Therefore, we set *ϕ* as 0.5%, 5% and 50%, and set *γ* as 1/14, 1/12, 1/10, 1/8, 1/6 and 1/4 to simulate the combined effect of SFTS under different intervention intensity.

The indicators of intervention effect included: cumulative number of cases, incidence rate, peak position of incidence and peak size of incidence. Finally, the model provided the optimal way to intervene in the spread of SFTS and provided guidance for the prevention and control of the outbreak of SFTS.

### Model simulation and statistical analysis

In this study, Excel was used for data summary and chart drawing, IBM SPSS Statistics (version 21.0.0.0) was used for data statistical analysis, Berkeley Madonna (Version 8.3.18) was used for fitting existing data and models, and epidemic prediction and evaluation were conducted for intervention effects under different transmission routes.

## Results

### Model fitting

**Fig. 4:**
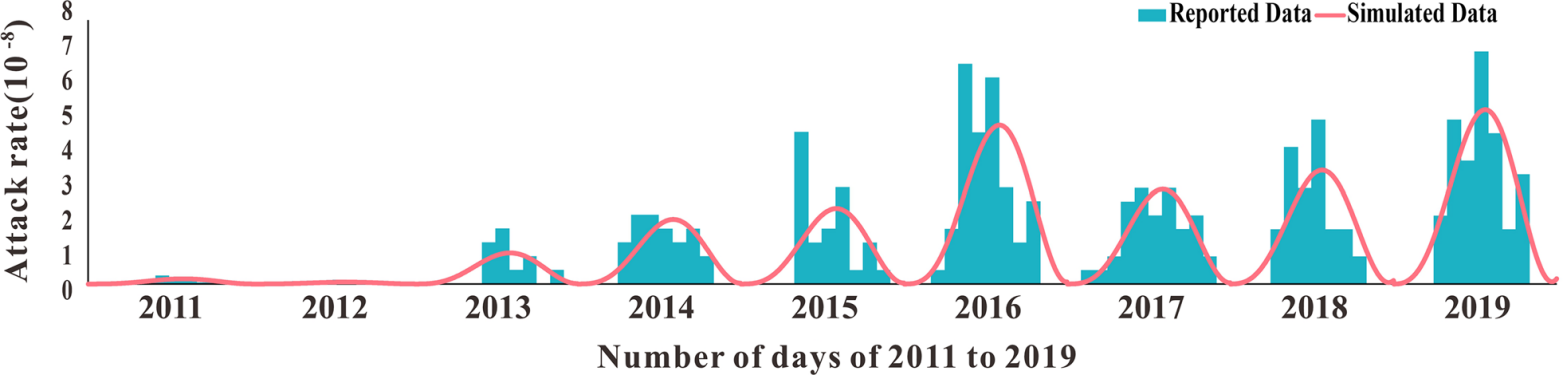
Model fitting results of the model to the reported SFTS incidence data from 2011 to 2019 in Jiangsu Province, China

The result of model fitting using 2011–2019 SFTS incidence rates in Jiangsu Province was shown in Fig. 4. By analyzing every month's and year's SFTS incidence rates, we discovered that the incidence rate of SFTS strictly changed seasonally. This meant that in a year, the incidence rate was high in summer and autumn with two peaks, and lowered with no cases in winter. Therefore, the seasonal correction function was added to carry on fitting in the model. Just like the results of our model fitting, the SFTS incidence rate of 2011–2019 in Jiangsu Province was periodically changed. It is often at rest in winter, begins to increase in spring and peaks by July–August. Notably, the peak incidence rate of SFTS in Jiangsu Province has increased to some extent in recent years compared with 2011–2014. The *R*^2^ and *P* values of the model fitting results for 2011–2019 are listed in Table 3. The model fitting results for all years were good, and *P* was significant (*P* < 0.05).

**Table 3:** Model fitting result of 2011–2019

Year	*R*^2^	*P*
2011	0.573878	< 0.05
2012	0.299520	< 0.05
2013	0.482155	< 0.05
2014	0.764983	< 0.05
2015	0.408163	< 0.05
2016	0.767604	< 0.05
2017	0.776788	< 0.05
2018	0.801787	< 0.05
2019	0.758567	< 0.05

### Model simulation of cutting transmission routes

Table 4 showed the simulation results of truncating different transmission routes based on the SFTS transmission dynamics model in Jiangsu Province in 2019. In general, when the cut transmission route has no effect, the cumulative number of cases was 671, and the peak time was 205 days.

**Table 4:** Interventional effect of cutting off different transmission routes on SFTS

“Knock-out” scenario*	Cumulative number of cases	Total attack rate	Peak time (days)	Peak attack rate
*β*_1_	0	0	0	0
*β*_3_	671	0	205	0
*β*_*w*_	0	0	198	0
*β*_21_	671	0	205	0
*β*_31_	671		205	0
*β*_23_	671	0	205	0
*β*_32_	671	0	205	0
*β*_3_*β*_*w*_	0	0	198	0
*β*_3_*β*_21_	671	0	205	0
*β*_3_*β*_31_	671	0	205	0
*β*_3_*β*_23_	671	0	205	0
*β*_3_*β*_32_	671	0	205	0
*β*_*w*_*β*_21_	0	0	198	0
*β*_*w*_*β*_31_	0	0	10	0
*β*_*w*_*β*_23_	0	0	198	0
*β*_*w*_*β*_32_	0	0	198	0
*β*_21_*β*_23_	671	0	205	0
*β*_21_*β*_31_	671	0	205	0
*β*_21_*β*_32_	671	0	205	0
*β*_31_*β*_23_	671	0	205	0
*β*_31_*β*_32_	671	0	205	0
*β*_23_*β*_32_	671	0	205	0
*β*_3_*β*_*w*_*β*_21_	0	0	198	0
*β*_3_*β*_*w*_*β*_31_	0	0	10	0
*β*_3_*β*_*w*_*β*_23_	0	0	198	0
*β*_3_*β*_*w*_*β*_32_	0	0	198	0
*β*_3_*β*_21_*β*_31_	671	0	205	0
*β*_3_*β*_21_*β*_32_	671	0	205	0
*β*_3_*β*_21_*β*_23_	671	0	205	0
*β*_3_*β*_31_*β*_23_	671	0	205	0
*β*_3_*β*_31_*β*_32_	671	0	205	0
*β*_3_*β*_23_*β*_32_	671	0	205	0
*β*_*w*_*β*_21_*β*_31_	0	0	10	0
*β*_*w*_*β*_21_*β*_23_	0	0	198	0
*β*_*w*_*β*_31_*β*_23_	0	0	10	0
*β*_*w*_*β*_21_*β*_32_	0	0	198	0
*β*_*w*_*β*_31_*β*_32_	0	0	10	0
*β*_*w*_*β*_23_*β*_32_	0	0	198	0
*β*_21_*β*_31_*β*_23_	671	0	205	0
*β*_21_*β*_31_*β*_32_	671	0	205	0
*β*_21_*β*_23_*β*_32_	671	0	205	0
*β*_31_*β*_23_*β*_32_	671	0	205	0
*β*_3_*β*_*w*_*β*_21_*β*_31_	0	0	7	0
*β*_3_*β*_*w*_*β*_21_*β*_23_	0	0	198	0
*β*_3_*β*_*w*_*β*_21_*β*_32_	0	0	198	0
*β*_3_*β*_*w*_*β*_23_*β*_32_	0	0	198	0
*β*_3_*β*_*w*_*β*_31_*β*_23_	0	0	10	0
*β*_3_*β*_*w*_*β*_31_*β*_32_	0	0	10	0
*β*_3_*β*_21_*β*_31_*β*_23_	671	0	205	0
*β*_3_*β*_21_*β*_23_*β*_32_	671	0	205	0
*β*_3_*β*_21_*β*_31_*β*_32_	671	0	205	0
*β*_3_*β*_31_*β*_23_*β*_32_	671	0	205	0
*β*_*w*_*β*_21_*β*_31_*β*_23_	0	0	10	0
*β*_*w*_*β*_21_*β*_31_*β*_32_	0	0	10	0
*β*_*w*_*β*_21_*β*_23_*β*_32_	0	0	198	0
*β*_*w*_*β*_31_*β*_23_*β*_32_	0	0	10	0
*β*_21_*β*_31_*β*_23_*β*_32_	671	0	205	0
*β*_3_*β*_*w*_*β*_21_*β*_31_*β*_23_	0	0	10	0
*β*_3_*β*_*w*_*β*_21_*β*_31_*β*_32_	0	0	10	0
*β*_3_*β*_*w*_*β*_21_*β*_23_*β*_32_	0	0	198	0
*β*_3_*β*_*w*_*β*_31_*β*_23_*β*_32_	0	0	10	0
*β*_3_*β*_21_*β*_31_*β*_23_*β*_32_	671	0	205	0
*β*_*w*_*β*_21_*β*_31_*β*_23_*β*_32_	0	0	10	0
*β*_3_*β*_*w*_*β*_21_*β*_31_*β*_23_*β*_32_	0	0	10	0

^*^“Knock-out” means to simulate cutting off the transmission routes by setting a parameter to 0, and estimates the contribution of the parameter by calculating the number of reduced cases or the total attack rate. The different scenario means which parameter was set as 0

We mainly studied the spread of SFTS in the population. When the transmission route between people *β*_1_ was cut off, SFTS cannot be transmitted in the population. With zero cases, the model cannot continue to simulate, and hence, other combinations containing *β*_1_ were not shown. When cutting off a single transmission route, it is worth noting that when the environmental transmission *β*_*w*_ was cut off alone, the number of cases also became zero. None of the other transmission routes cut off alone reduced the number of SFTS infections. When two transmission routes, such as a combination with environmental transmission were cut off, the number of patients was also reduced. When the environmental transmission *β*_*w*_ and animals-to-humans transmission *β*_31_ were cut off together, the peak time of disease was advanced to 10 days. When other combinations that can cause a decrease in SFTS cases were cut off, the peak time was advanced to 198 days. When three transmission routes (such as the combination of environmental transmission *β*_*w*_, animals-to-humans transmission *β*_31_ and ticks-to-humans transmission *β*_21_) were cut off at the same time, the number of cases decreased significantly. And similarly, as above, when *β*_31_ and *β*_w_ were cut off, the peak time was advanced to 10 days. With a cut-off of environmental transmission *β*_*w*_ and animals-to-ticks transmission *β*_32_, ticks-to-animals transmission *β*_23_ and ticks-to-humans transmission *β*_21_ at the same time, the peak time was also increased to 198 days. When four transmission routes were cut off at the same time (such as *β*_3_, *β*_*w*_, *β*_21_ and *β*_31_), the peak time of SFTS was advanced to 7 days. The remaining results were the same as above. When five and six transmission routes were cut off at the same time, SFTS only occurred when environmental transmission was not cut off, and the peak onset time was not advanced. For more details, see Table 4.

### Taking different interventions

In the study of separate interventions, the shortening of the disease course was assumed to be no shortening and shortening of 2 days, 4 days, 6 days, 8 days and 10 days; the isolation ratio was assumed to be: 98%, 90%, 80%, 70%, 60% and 50%. When studying the combined effects of the two interventions, the isolation ratios were assumed to be 0.5%, 5% and 50%. It turned out that when a separate intervention was implemented alone, shortening the course of the disease will not limit the spread of fever with thrombocytopenia syndrome. But when the quarantine was implemented, only one case was reduced. When the joint intervention was implemented (i.e., the course of the disease was not shortened and the isolation ratio was 5% and 50%), only one case was reduced. Therefore, these two interventions had little effect on the number of cases of fever with thrombocytopenia syndrome, as shown in Tables 5 and 6.

**Table 5:** Interventional effect of taking joint interventions

Intervention	Cumulative number of cases	Total attack rate	Peak time (days)	Peak attack rate
*γ* = 1/14 + *∅* = 0	530	0.00	197	0
*γ* = 1/14 + *∅* = 0.5%	531	0.00	197	0
*γ* = 1/14 + *∅* = 5%	530	0.00	197	0
*γ* = 1/14 + *∅* = 50%	530	0.00	197	0
*γ* = 1/12 + *∅* = 0	531	0.00	197	0
*γ* = 1/12 + *∅* = 0.5%	531	0.00	197	0
*γ* = 1/12 + *∅* = 5%	531	0.00	197	0
*γ* = 1/12 + *∅* = 50%	531	0.00	197	0
*γ* = 1/10 + *∅* = 0	531	0.00	197	0
*γ* = 1/10 + *∅* = 0.5%	531	0.00	197	0
*γ* = 1/10 + *∅* = 5%	531	0.00	197	0
*γ* = 1/10 + *∅* = 50%	531	0.00	197	0
*γ* = 1/8 + *∅* = 0	531	0.00	197	0
*γ* = 1/8 + *∅* = 0.5%	531	0.00	197	0
*γ* = 1/8 + *∅* = 5%	531	0.00	197	0
*γ* = 1/8 + *∅* = 50%	531	0.00	197	0
*γ* = 1/6 + *∅* = 0	531	0.00	197	0
*γ* = 1/6 + *∅* = 0.5%	531	0.00	197	0
*γ* = 1/6 + *∅* = 5%	531	0.00	197	0
*γ* = 1/6 + *∅* = 50%	531	0.00	197	0
*γ* = 1/4 + *∅* = 0	531	0.00	197	0
*γ* = 1/4 + *∅* = 0.5%	531	0.00	197	0
*γ* = 1/4 + *∅* = 5%	531	0.00	197	0
*γ* = 1/4 + *∅* = 50%	531	0.00	197	0

^a^*γ* represent the intervention of shorten disease course and ϕ represent the proportion of isolation

**Table 6:** Interventional effect of taking isolation

Isolation ratio	Cumulative number of cases	Cumulative attack rate	Peak time (days)	Peak attack rate
50%	530	0.00	201	0.00
60%	530	0.00	201	0.00
70%	530	0.00	201	0.00
80%	530	0.00	201	0.00
90%	530	0.00	201	0.00
98%	530	0.00	201	0.00

## Discussion

SFTS is an emerging infectious disease caused by SFTSV. Recently, the incidence rate has been increasing year by year, and the coverage of the epidemic area has expanded. Jiangsu Province is one of the provinces with the highest incidence of SFTS, and they have conducted stringent SFTS monitoring. This study was established to use a model based on the SFTS monitoring data of Jiangsu Province from 2011 to 2019. The SFTS transmission dynamics model was used to simulate the change in incidence rate by cutting different transmission routes, and taking interventions into consideration and assessing the effect of those interventions.

### The effect of knock-out simulation

Previous studies have shown that the number of SFTS patients infected by the environment were more than those who were infected by ticks [19, 30–33]. But most studies only use the density of ticks in the environment and the antibody of SFTSV in ticks to evaluate the hazardous situation of the environment [22, 48–50], so they did not consider the environment as one of the transmission route. As we all know, several factors such as the number of ticks, the dominant phase of ticks, outdoor activities and the change of weather can affect the occurrence of SFTS [51]. There was one study that collected SFTSV from inanimate objects in the room of SFTS patients. Thus, the environment could be considered as one of the transmission routes of SFTS, although a test result of the air sample was negative [52]. Therefore, based on the literature and the 2019 data of SFTS incidents in Jiangsu Province, we established the SFTS transmission dynamics model and used the coefficient of transmissibility to reflect the different routes of transmission. We simulated cutting different transmission routes to show the different effects of interventions. The result of this study showed that in all the combinations of cutting different transmission routes, the cumulative number of SFTS cases and other indicators could be significantly reduced when the transmission route containing the environment was cut off. Moreover, the intervention effect was more obvious when the environmental and host animal transmission routes were cut simultaneously. Those results indicated that the environment is an important route for the transmission of SFTS, and this was consistent with findings of previous research. Besides, this also provided a direction that can be explained as follows. It is possible that people were not exposed to a wild environment, and the chance of contacting ticks or animal excreta, blood, etc. decreased and accounted for the low SFTS incidence rate. In addition, some literatures have shown that SFTS was an environment-tick-host animal-tick cycle and that humans were infected by accidental participation in one of the steps. Therefore, the more transmission ways cut off, the smaller the impact of SFTS was. Interestingly, the effect of cutting off the environment and the transmission route of ticks on SFTS was less than that of cutting off the environment and host animals simultaneously. This might be the main way for people to be infected with SFTS except for tick bites. In previous studies, there were also literatures showing that those infected with SFTS with a clear history of tick bites only accounted for a small proportion (about 22–29% [6, 33, 53]) of all infected individuals. The study also suggested that humans might be infected with SFTS after exposure to animal blood [6]. Although the transmission of SFTS was initiated by ticks, it may be that the two transmission routes of animal and environment are more important for human beings.

### The effect of taking interventions

For infectious diseases, there are three links and two factors. The three links are: the source of infection, transmission routes and the population. The two factors include natural factors and social factors. Common measures to control infectious diseases include controlling the source of infection, cutting off the route of transmission and protecting the population. Therefore, after cutting off the route of transmission and protecting the population, there is still a need to control the source of infection. In that regard, we chose to take interventions including taking isolation and shortening the course of the disease. We also assessed the effects of taking isolation and shortening the course of the disease according to the established SFTS transmissibility model. Based on the data of SFTS, the results of the simulation were used to evaluate the effect of the two interventions on SFTS. The results showed that whether implementing isolation alone or shortening the course of the disease, or combining the two interventions, the effect of interventions mentioned above on the incidence of SFTS was limited. And the different isolation ratio had little effect on the cases. This may be because that the incidence of SFTS is low. People living in cities were less likely to be infected with SFTSV as most of the patients are farmers or field workers [19, 32, 33, 51]. Even with humans-to-humans transmission, the greatest chance of occurrence was only among members of the population who come in contact with blood, such as nurses or family members [54]. In addition, secondary SFTS infection leads to milder clinical manifestations compared with primary SFTS. Therefore, secondarily infected patients only experience sudden fever, thrombocytopenia, leukopenia and gastrointestinal symptoms and recover more quickly [55].

In summary, implementing isolation and shortening the course of the disease had little impact on SFTS. This may suggested that prevention and control of the development of SFTS should focus on cutting off the direction of transmission routes. Therefore, according to the results of cutting off the transmission route earlier, interventions to prevent the transmission of SFTS suggest the following several aspects: First, for the environment, a long stay in the wild environment should be avoided. Also, residential areas should be cleaned/sanitized regularly and field working personnel should be equipped with protective clothing and gear such as insect repellents. Secondly, regarding tick transmission, regular repellent work should be done in rural and suburban areas to prevent tick breeding. Third, for animal transmission, the poultry and other domesticated animals should be well protected in rural areas to avoid the livestock being bitten by infected wild animals. At the same time, the livestock should be regularly treated with insecticide, physically examined for ticks and their serum should be tested regularly for SFTSV. Finally, for human-to-human transmission, medical staff and patients’ families should protect themselves when handling SFTS cases and avoid direct contact with the patient’s body or blood. Hospitals and township health centers in high-risk areas should be equipped with corresponding protective gear for use. Additionally, as our research results showed that isolation of SFTS patients or shortening the course of the disease cannot reduce the spread of SFTS, we should reduce the formulation of such plans to avoid wasting resources.

## Limitations

There were some limitations in our study: first, the data of SFTS cases collected by the China Information Network System of Disease Prevention and Control. Since the capability and availability of this system was finite, our findings need to be interpreted with caution. Second, this study failed to separate the effects of ticks and host animals on humans in the environment. Third, some parameters of our model were collected from literature that may represent certain error in terms of timeliness.

## Conclusions

In this study, the transmission dynamics model of SFTS was constructed based on the epidemiological characteristics of SFTS in Jiangsu Province. The effects of cutting off different transmission routes and taking isolation and shortening the course of the disease on SFTS control were evaluated. Finally, it was determined that cutting off the transmission route from the environment to humans had the greatest effect on SFTS prevention and control.

## Supplementary Information

**Additional file 1: Figure S1.** The location of Jiangsu Province in China.

## Data Availability

Data supporting the conclusions of this article are included within the article.

## Abbreviations

SFTS: Severe fever with thrombocytopenia syndrome
SFTSV: Severe fever with thrombocytopenia syndrome virus
SEIAR: Susceptible–exposed–infectious–asymptomatic–recovered
MMDM: Multi-population and multi-route dynamic model
SEA: Susceptible–exposed–asymptomatic
CDC: Centers for Disease Control and Prevention
